# A17 ROLE OF SEX-SPECIFIC GUT EPITHELIAL α2,6-SIALYLATED GALACTOSE IN COLONIC MUCUS FUNCTION, MICROBIAL ECOLOGY, AND PROTECTION FROM COLITIS.

**DOI:** 10.1093/jcag/gwae059.017

**Published:** 2025-02-10

**Authors:** K Bergstrom, M Melvin, S Jafaripour, N Fancy, J Irungu, P Daneshgar, R Inaba, G Hans, c ma, Q Liang, B vallance, W Zandberg

**Affiliations:** The University of British Columbia Okanagan, Kelowna, BC, Canada; The University of British Columbia Okanagan, Kelowna, BC, Canada; The University of British Columbia Okanagan, Kelowna, BC, Canada; The University of British Columbia Okanagan, Kelowna, BC, Canada; The University of British Columbia Okanagan, Kelowna, BC, Canada; The University of British Columbia Okanagan, Kelowna, BC, Canada; The University of British Columbia Okanagan, Kelowna, BC, Canada; The University of British Columbia Okanagan, Kelowna, BC, Canada; The University of British Columbia, Vancouver, BC, Canada; The University of British Columbia, Vancouver, BC, Canada; The University of British Columbia, Vancouver, BC, Canada; The University of British Columbia Okanagan, Kelowna, BC, Canada

## Abstract

**Background:**

The heavily glycosylated Mucin-2 (MUC2) comprises gut mucus, which protects the colon from microbially-induced inflammation. Over 100 unique oligosaccharides (glycans) are found on MUC2, contributing to its barrier and microbiota-modulating functions. A major sugar on MUC2 glycans is sialic acid (Sia), which can occur via different stereoisomeric linkages, including an α2,6 linkage to galactose (Siaα2,6Gal). Siaα2,6Gal is a capping structure on mucus glycans that interacts with the microbiota. Synthesis of Siaα2,6Gal epitopes is mediated by beta-galactoside alpha-2,6-sialyltransferase 1 (ST6Gal1), and is implicated in sex-specific pathologies. However, its impact on gut mucus, microbiota, and colitis, along with the degree to which this is sex-specific, is unknown.

**Aims:**

The aim of this study was to determine if the loss of gut epithelial Siaα2,6Gal has a sex-specific impact on gut mucus, microbiota and susceptiblity to colitis.

**Methods:**

To address our aim, we generated mice conditionally deficient in ST6Gal1 in intestinal epithelial cells (IEC) using CreLox technology (IEC *St6gal1-/-* mice). We compared “floxed” male and female *St6gal1f/f* (wild-type, WT) and IEC *St6gal1-/-* littermates, focusing on mucus structure *in situ*, mucin glycomics via High-Performance Liquid Chromatography-Mass Spectrometry (HPLC-MS), and microbiota composition through 16S rRNA profiling and metabolomic analysis. Clinical disease activity and histologic scores of colitis were assessed at baseline and following exposure to chemical (2.5% Dextran Sodium Sulfate, DSS) and microbial (*Citrobacter rodentium*) agents.

**Results:**

Lectin staining confirmed a complete loss of Siaα2,6Gal in the colon crypts of IEC *St6gal1-/-* mice, validating the functionality of our model. At baseline, no major clinical symptoms or differences in mucus barrier integrity were observed between WT and IEC *St6gal1-/-* mice of either sex. However, sialomic and glycomic analyses revealed sex- and genotype-specific differences, with a notable impact on the relative mucin sulfation status in female IEC *St6gal1-/-* mice. Microbiota profiling highlighted an increased abundance of *Bifidobacterium* spp. in WT mice, a higher proportion of certain *Firmicutes* members in male IEC *St6gal1-/-* mice, and sex-specific differences in short-chain fatty acid production. DSS-induced colitis showed mildly worsened disease outcomes in male IEC *St6gal1-/-* mice, while acute bacterial-induced colitis caused by *C. rodentium* led to more severe large-intestinal inflammation in female IEC *St6gal1-/-* mice.

**Conclusions:**

This work expands the current knowledge of the role of Siaα2,6Gal in vivo and its sex-specific impact on overall glycosylation, microbial ecology, and colitis susceptiblity.

**Funding Agencies:**

CCC

